# The irreconcilability of insight

**DOI:** 10.1007/s10071-024-01844-y

**Published:** 2024-03-02

**Authors:** Eli Shupe

**Affiliations:** University of Texas at Arlington, Arlington, USA

**Keywords:** Insight, Problem-solving, Causal reasoning, Means-end reasoning, Gestalt psychology

## Abstract

We are said to experience insight when we suddenly and unexpectedly become aware of the solution to a problem that we previously took ourselves to be unable to solve. In the field of comparative cognition, there is rising interest in the question of whether non-human animals are capable of insightful problem-solving. Putative cases of animals demonstrating insight have generally attracted two types of criticism: first, that insight is being conflated with other cognitive capacities (e.g., causal cognition, or mental trial and error); and, second, that the relevant performances merely reflect associative learning—and on the received understanding of insight within comparative cognition, insight necessarily involves non-associative processes. I argue that even if we grant that some cases of animal insight do withstand these two criticisms, these cases of purported animal insight cannot shed light on the nature of insightful problem-solving in humans. For the phenomenon studied by cognitive psychologists under the heading of insight is fundamentally different from that studied in comparative cognition. In light of this impasse, I argue that the reinterpretation of the extant research on animal insight in terms of other high-level cognitive capacities (means-end reasoning in particular) can improve the prospect of a successful comparative research program.

## Introduction

Insight is said to occur when we abruptly and unexpectedly grasp a solution to a problem, often by way of a radical shift in our representation of the problem or its components (Cushen and Wiley 2012; Sternberg and Davidson 1995; Metcalfe and Wiebe 1987; Weisberg 1995; Wertheimer 1945). Insight is also strongly associated with (and sometimes even defined fundamentally in terms of) its distinctive phenomenological character, the pleasurable ‘eureka experience’ or ‘aha moment’ that accompanies the arrival of an insightful solution (Bowden et al. 2005; Duncker 1945; Metcalfe and Wiebe 1987).

Comparative psychologists, who study the mental processes of non-human animals, have taken up the question of whether nonhuman animals also engage in insightful problem-solving. Scientists working in this research program claim to have found evidence of insight in elephants (*e.g.*, Foerder et al. 2011), various great apes (*e.g.,* Hanus et al. 2011; Mendes et al. 2007), and several species of birds (*e.g.*, Bird and Emery 2009a; Bird and Emery 2009b; Huber and Gajdon 2006; Pepperberg 2004). Because comparative psychologists typically understand insightful performances in non-human to involve non-associative mental processes, these findings have been held up as evidence of sophisticated higher reasoning abilities in nonhuman animals. These claims have drawn a great deal of positive attention, but also some criticism: skeptics contend that much of this work fails to successfully rule out competing deflationary explanations according to which the behaviors in question are merely the products of associative learning rather than genuine cases of insightful problem-solving (Epstein et al. 1984; Heyes 2012; Shettleworth 2012). As a result, work on insight in nonhuman animals is primarily engaged in the search for exemplars of non-associative problem-solving that are resistant to skeptical debunking.

I shall argue, however, that successes along these lines come with substantial caveats. Even if some instances of nonhuman problem-solving genuinely are the products of non-associative mental processes, there are deeper worries about classifying these performances as *insightful*. At best, to do so is premature; at worst, it is misleading. In what follows, I will show that a great deal more conceptual work needs to be done before we can assert that insightful problem-solving is a capacity that humans and nonhuman animals share. This is because there is a serious tension between how researchers studying humans and those studying nonhuman animals characterize, operationalize, and ultimately understand insight and insightful problem-solving.

## Can animal insight teach us about ourselves?

Some comparative psychologists work to understand the thought and behaviour of just one particular animal family, genus, or even species. Others seek out truths that are common across various kinds of creatures, hoping to better piece together their intertwined phylogenetic histories. Still others focus on the commonalities and differences between humans and nonhuman animals. Such researchers harness discoveries about nonhuman animals to increase our understanding of human cognition, thus contributing to and working in tandem with the general project of cognitive psychology. My interest in this paper lies in whether the study of insight in nonhuman animals can contribute to this particular goal.

How might the study of insight in nonhuman animals deepen our understanding of the same phenomenon in humans? First, if insight is not a uniquely human phenomenon, by discovering it in nonhuman animals we might learn something about its phylogenetic origins. Perhaps we can inform our guesses about when, in our own evolutionary history, we developed the capacity for insightful problem-solving. Furthermore, if we discover it in species that bear little close relation to *homo sapiens*, then this evidence of convergent evolution can reveal the kinds of evolutionary pressures that are conducive to the development of the capacity.

Second, discoveries about insight in nonhuman animals have the potential to constrain our theories about the cognitive underpinnings of insight in general. We might come to discover which processes or capacities are necessary or sufficient for the occurrence of insightful problem-solving and which are not. Consider an example. Say that we agree that creatures that do *φ* demonstrate insight, and we observe both humans and domestic cats *φ*ing. By our lights, then, both humans and cats would be capable of insight. Based on this evidence, we could rule out theories on which the cognitive processes underlying insight necessarily rely on the presence of sophisticated, language-like representations, as it is unlikely that domestic cats possess such mental representations.

So it is clear, as the many psychologists pursuing such research would surely agree, that there is much that could be learned from a successfully comparative study of insight in humans and nonhuman animals. We can specify three requirements for success for a research program of this kind:

1. We must be able to identify cases of insight in humans.
2. We must be able to identify cases of insight in nonhuman animals.
3. These cases of insight must plausibly be exercises of the same capacity, based on criteria that are not arbitrarily disjunctive.

While perhaps not sufficient, at the very least these are necessary prerequisites: a comparative investigation of insight in humans and nonhuman animals must satisfy all three of the conditions above if it is to be a productive scientific enterprise. (1) and (2) are relatively straightforward to explain. (1) requires that the psychologists studying insight in humans have agreed-upon performance measures for determining when, by their lights, a human has solved a problem insightfully. Likewise, (2) requires that the comparative psychologists have agreed-upon performance measures for determining when, by their lights, a nonhuman animal has solved a problem insightfully. Finally, condition (3) simply requires that we are talking about roughly the same thing when we talk about insight in humans and insight in nonhuman animals.

In this paper, I shall argue that many of the attempts to provide a unified account of insight in humans and nonhuman animals have failed to satisfy this third criterion. To set up my arguments to that effect, however, I first must discuss how psychologists have taken themselves to satisfy conditions (1) and (2). In the next section, I will discuss what researchers working on insight humans and nonhuman animals each mean by insight, and how they operationalize that notion to identify its instances. We must have a clear picture of the relevant concepts of insight that are deployed in these literatures before we can assess the possibility of integrating them.

## Insight in humans and nonhuman animals

### Insight in humans

In cognitive psychology, ‘insight’ most commonly refers to the sudden and unexpected awareness of the solution to a difficult problem, often involving a radical reconceptualization of the solver’s understanding of the problem’s components or constraints (Sternberg and Davidson 1995; Metcalfe and Wiebe 1987; Weisberg 1995; Wertheimer 1945). Most researchers think of insight as importantly distinct from conventional analytic problem-solving (although see Maltzman 1955, Thorndike 1911, and Weisberg 1988 for arguments otherwise).

**Fig. 1:**
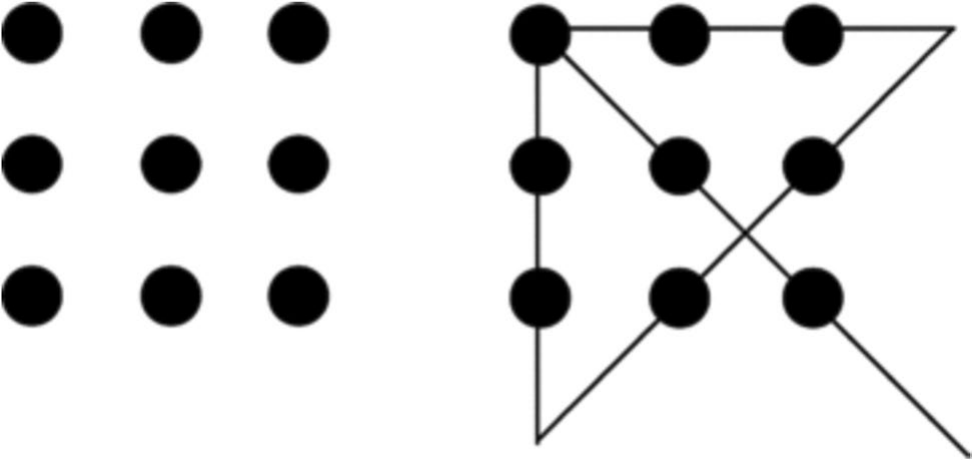
The nine-dots problem

In experimental work, insightful problem-solving in humans is often investigated by using visual puzzles, particularly ones whose solutions require ‘lateral thinking’ on the part of the solver. Perhaps the most frequently used test for insight in human subjects is the nine-dots problem, shown in Fig. 1 alongside its solution (Chronicle et al. 2001; Kershaw and Ohlsson 2004; MacGregor et al. 2001; Maier 1930; Öllinger et al. 2014). Given the dot array on the left, a solver is told that she must connect all nine dots without lifting her pencil from the paper, using only straight lines, and using no more than four strokes. As illustrated below, the solution requires the solver to realize she can extend her lines beyond the square ‘box’ formed by the dots.

Typical, spontaneous solutions of problems of this kind are said to involve insight on the part of the problem-solver, at least in cases where the solver is not simply drawing from previous experience with such puzzles and their solutions. To rule out this experience-based, non-insightful solution path, experimenters rely heavily on their participants’ verbal descriptions of their problem-solving experiences. In particular, ‘eureka’ or ‘aha’ phenomenology is often taken to be diagnostic or even definitional of insight (cf. Kaplan and Simon 1990).

For a more comprehensive discussion of experimental paradigms used to study insight in humans, interested readers can see Batchelder & Alexander (2012), Chu and MacGregor (2011), Öllinger and Knoblich (2009), Sternberg and Davidson (1995), all of which contain detailed reviews.

There are three broad features in terms of which insight in humans tends most commonly to be characterized. I will discuss them roughly in the order of their level of acceptance in the literature.

1. Psychological impasse
  Psychological impasse often precedes insightful problem-solving (Bowden et al. 2005; Kaplan and Simon 1990; Ohlsson 1992; Schooler 1995). The duration of this stage varies, ranging from a fleeting moment to an extended period of days, weeks, and even years, during which the solver may or may not be actively concentrating on the problem in question (Mayer 1995). Problem-solvers become ‘stuck’ prior to insightful realizations for a variety of reasons, such as *mental set*, the predisposition to tackle problems using methods that proved successful with similar-seeming problems in prior experience (Öllinger et al. 2014), or *functional fixedness*, whereby the conventional use of an object causes the solver to overlook other salient features, such as material or shape, which might be more instrumental to solving the problem at hand (Duncker 1945; Ohlsson 1992).
  Whether insight *necessarily* involves prior psychological impasse is debatable. Recently, Hans Stuyck and his co-authors (2021) have compellingly argued that the “full-blown experience of impasse” is less frequently associated with insight than previously thought, though they concede that some “more subtle form of impasse” such as early cognitive conflict detection may still play an important precursor role.
2. Mental restructuring
  Insightful problem-solving is typically thought to involve a cognitive change or shift in how one represents or reasons about the problem or its components (Becker et al. 2021; Ohlsson 1984; Öllinger et al. 2014; Weisberg 1995; Wills et al. 2006). The Gestalt psychologists likened it to the abrupt perceptual shifts experienced when viewing ambiguous visual figures, such as the Necker cube (Köhler 1957; Necker 1832; Schooler et al. 1995). This mental restructuring has been understood differently across the literature (Weisberg 1995). Some take it to involve shifts of attentional focus among the elements of a problem (Becker et al. 2021; Knoblich et al. 1999; Kounios & Beeman 2014; Ohlsson 1992); others as involving novel conceptual recombinations (Boden 2004; Koestler 1964); and many as involving the reinterpretation or relaxation of the problem’s understood constraints (Chu and MacGregor 2011; Knoblich et al. 1999; Metcalfe and Wiebe 1987; Ohlsson 1992; Weisberg 1995; Wertheimer 1945).
3. Distinctive phenomenology
  As Sara Shettleworth puts it, “the acid test of insightful problem solution in people is its distinctive phenomenology, the ‘aha’ experience”, whereby the solution seems to reveal itself in a surprising and often gratifying manner (Duncker 1945; Gick and Lockhart 1995; Gruber 1995; Kaplan and Simon 1990; Metcalfe and Wiebe 1987; Schooler et al. 1993; Shettleworth 2012; Topolinski and Reber 2010). Rather than the problem-solver making stepwise progress toward an insightful solution, they will typically become consciously aware of the solution (or means of deriving it) all at once (Gick and Lockhart 1995; Metcalfe and Wiebe 1987; Schooler et al. 1993; Topolinski and Reber 2010). Some researchers also discuss the insightful problem-solver’s high sense of confidence or certainty as an important part of insight’s phenomenological profile (*e.g.*, Gruber 1995; Ross and Vallée-Tourangeau 2022, Weisberg 2013).
  It is hard to overstate the weight that insight’s phenomenological character has been given in the literature on insightful problem-solving. As Kaplan and Simon (1990) note, “[a]lthough numerous definitions of insight have been offered … many researchers consider the subjective AHA! feeling to be a critical component” (and indeed, they themselves “use insight to refer to a subjective AHA! Experience during problem solving”). Metcalfe & Wiebe, similarly, have proposed “that the difference in phenomenology accompanying insight and noninsight problem solving … be used to define insight” (Metcalfe and Wiebe 1987). It is not surprising, then, that experimental tests for and of insight in humans rely extensively on phenomenological self-report.

### Insight in nonhuman animals

I will now discuss how those studying the problem-solving abilities of nonhuman animals have understood and operationalized the concept of insight. Here, it bears being explicit that most comparative psychologists who study insight in nonhuman animals *do* take themselves to be studying the same phenomenon as that which occurs in humans. Often, for example, they introduce the idea of insight to their readers by way of reference to the ‘aha!’ moment, and other aspects of insight’s distinctive phenomenology that we are familiar with from human experience (*e.g.,* Neilands et al. 2016; Renner et al. 2017). Alice Auersperg, in one discussion of avian creativity, goes so far as to explicitly state that “[t]he amazing abilities being shown by these birds are demonstrations of their successful completion of what are termed in the human literature as ‘insight problems’”, with “[w]ell-known examples of problems in this category include[ing] the ‘Nine Dot’ and Duncker’s Candle tasks” (Auersperg 2015).

The fact that the presence of ‘aha’ phenomenology is the typical diagnostic test for insight in humans, however, complicates the study of insight in nonhuman animals (Shettleworth 2012). Because their subjects are incapable of verbal report, those who work with nonhuman animals must advert to other means of identifying insightful problem-solving. It is unsurprising, then, that experiments studying insight in nonhuman animals differ greatly from experiments investigating insight in humans.

With some exceptions, the experimental paradigms used in studies of insight in nonhuman animals can be categorized into four groups: (1) **string-pulling**, where an out-of-reach reward is accessed by pulling an attached string, often with various added complexities (Jacobs and Osvath 2015; Wasserman et al. 2013); (2) **use of object to access reward**, such as in Köhler’s box-stacking experiment, where use of said object may or may not also amount to tool use (Köhler 1957). A sophisticated version of this paradigm is what Neilands et al. (2016) have called the **Von Bayern paradigm**, where stones are dropped to collapse a platform on which there is an out-of-reach reward^1^; (3) the **Aesop’s fable paradigm**, where subjects must obtain a floating reward from a deep, narrow container by adding or displacing water; and, lastly, (4) **tool-making** and **metatool-use**, where subjects, respectively, either construct a tool to access a reward or use tools to access other tools with which a reward can be obtained.

Table 1 below lists some notable reports of or experiments on insightful problem-solving in nonhuman animals, as well as the paradigm to which they adhere, and quotations that illuminate their respective definitions of insight.

**Table 1:** Insight in nonhuman animals

Year	Author(s)	Title	Species	Paradigm	Account of insight
1925	Köhler	*The Mentality of Apes*	Chimpanzee	Use of object to access reward; string-pulling	“Hence follows this criterion of insight: the appearance of a complete solution with reference to the whole lay-out of the field.”; “A total field would be experienced without insight if all its several states, wholes, attitudes etc. were simply given as a pattern in which none was felt directly to depend upon any other and none to determine any other.”
1925	Yerkes (in Yerkes & Learned)	*Chimpanzee Intelligence and its Vocal Expression*	Chimpanzee	Use of object to access reward	“The suddenness of Chim’s success in the box stacking experiment suggests the orangutan’s solution of a multiple choice problem. The animal, after many days of effort which seemingly brought the solution no nearer, suddenly achieved success. Seemingly the problem was solved over-night. The only reasonable explanation of such sudden and radical change in behavior is insight. Köhler has described similar behavior in adolescent chimpanzees.”
1927	Yerkes	*The Mind of a Gorilla*	Gorilla	Use of object to access reward	“trial of more or less adequate mode of response,”, “hesitation, pause, attitude of quiet concentration”, “appearance of [a] critical point at which the organism suddenly, directly and definitely performs required adaptive act", “ready repetition of adaptive response after once performed.” (quoted in Beck 1967)
1929	Maier	Reasoning in white rats	Rat	Other (taking novel shortcut in maze)	"[Insight consists of] the ability to bring together spontaneously two elements of past experience without having them previously associated by contiguity"; “[T]he rat first responded in a random ‘trial and error’ fashion, then he suddenly changed to a ‘purposeful’ form of behavior and thereafter continued in such behavior, which the author considers indicative of ‘insight.’”
1933	Bierens De Haan	Der Stieglitz als Schöpfer [Creative ability in the goldfinch]	Goldfinch	String-pulling	Exegesis from Thorpe (1943): “The great individual variation in ability shown by Bierens de Haan’s Goldfinches and the improbability of the action being in any way appropriate to the life of the bird under natural conditions forced that author to the conclusion that such behaviour cannot regarded as instinctive in the sense of being an inborn automatism. He therefore regards it as individual learning involving, at least in part, genuine insight.”
1937	Grether & Maslow	An experimental study of insight in monkeys	Monkey (various species)	Other (disjunctive syllogism)	“… the solution of … a problem by involving the synthesis of two separate situations”; “Mechanical principles, such as ‘trial-and-error’ and ‘conditioning,’ do not appear adequate to explain the 3 manners of attaining success on the problem.”
1943	Thorpe	A type of insight learning in birds	Great tit	String-pulling	“ … the act had every appearance of ‘insight learning’ in the sense used by W. Köhler in his classic work *The Mentality of Apes*, a real solution of a problem, distinguishing means and ends, based on apprehension of the essential relations in a situation and not upon the slow method of ‘Trial and Error’ which consists of profiting by accidental ‘discovery,’ and in gradually eliminating useless or unsuccessful actions.”
1945	Thorpe	Further notes on a type of insight learning in birds	Various species	String-pulling	Follow-up to previous article; “… insight of the same type and degree as that described in my own article.”
1945	Birch	The relation of previous experience to insightful problem-solving	Chimpanzee	Use of object to access reward	“By insight, [Köhler (1925)] meant a type of behavior in which the animal takes the meaningful functional relations existing in the whole situation into account, and performs in a continuous, sequential manner until a goal is achieved.”
1956	Thorpe	*Learning and Instinct in Animals*	Various	Various; survey of previous research. Included in this table because of its theoretical influence on the literature	“the sudden production of a new adaptive response not arrived at by trial behavior”; “the solution of a problem by the sudden adaptive reorganization of experience.”
1956, 1958, 1961	Vince	“String pulling” in birds I (1956), II (1958), & III (1961)	Various avian species	String-pulling	“More recently Thorpe (1956, pp. 335–9) has discussed observations on a large number of birds showing differences between species and between individuals of the same species. Thorpe suggests […] that since the food is obtained by manipulation of the string the behaviour may imply ‘insight’ into the food-string relationship (trial and error, or an inborn behaviour pattern may, however, contribute towards it)”; “The apparatus described by Thorpe (1956) and a similar procedure were used.”
1984	Epstein, Kirshnit, Lanza, & Rubin	‘Insight’ in the pigeon: antecedents and determinants of an intelligent performance	Pigeon	Use of object to access reward	“The suddenness, directness and continuousness of the performances satisfy Köhler’s criteria for ‘genuine’ or ‘insightful’ solutions.”
1995	Heinrich	An experimental investigation of insight in common ravens (Corvus corax)	Raven	String-pulling	“According to Webster’s dictionary, insight is defined as ‘the power or act of seeing into a situation’”; “Insight can be shown indirectly to play a role in a behavior where learning and/or responses present from birth can be eliminated”
2004	Pepperberg	“Insightful” string-pulling in Grey parrots (Psittacus erithacus) is affected by vocal competence	Grey parrot	String-pulling	“The ability, without training, to obtain food suspended by a string by reaching down, pulling up a loop of string onto the perch, stepping on the loop to secure it, and repeating the sequence several times (e.g., to demonstrate an understanding of intentional means-end behavior; see review in Willatts 1999) has been used to assess ‘insight’ in avian species.”; “…this test of insight…”
2003	Hihara, Obayashi, Tanaka, & Iriki	Rapid learning of sequential tool use by macaque monkeys	Japanese macaque	Tool-making or meta-tool-use	“It has been suggested that chimpanzees have an ‘insight,’ which leads to a sudden appearance of a complete and correct response to a complex tool problem (Köhler 1925), while a solution learned through trial-and-error or associative learning occur[s] gradually. The presently shown rapid mastering of a complex stick task in monkeys would involve a distinct learning process different from the mastering of simple stick tasks through slowly emerging trial-and-error learning process […]. [T]he former might involve a mental component possibly correlative to an insightful process described above.”
2006	Huber & Gajdon	Technical intelligence in animals: the kea model	Kea	String-pulling	“Thorpe (1956) proposed that insight, on the one hand, should be considered primarily as a matter of the organization of perceptions, leading to the apprehension of relations. Insight learning, on the other hand, includes as an essential element the appropriate organization of effector response, and can be defined as ‘the sudden production of a new adaptive response not arrived at by trial behaviour or the solution of a problem by the sudden adaptive reorganization of experience’ (Thorpe 1956, p 100).”; “We interpreted their performance as insightful in the sense of being sensitive of the relevant functional properties of the task and thereby producing a new adaptive response without trial-and-error learning.”;
2006	Werdenich & Hubner	A case of quick problem solving in birds: string pulling in keas, Nestor notabilis	Kea	String-pulling	“Instead, there is increasing evidence that the apprehension of cause–effect relationships between two or more physical objects, including the subject’s own body, guides the covert assembly of the response sequence, the exact manner in which objects are manipulated, and which of several alternative objects are used (Hauser, 1997, Heinrich 2000, Rumbaugh et al*.*., 2000). Perhaps this is what Thorpe (1956) called insight in contrast to trial-and-error learning, in which actions are selected through reinforcement. We believe that our keas’ string-pulling behaviour can be best explained by such apprehension of a cause–effect relationship and the adaptive reorganization of experience.”
2007	Mendes, Hanus, & Call	Raising the level: orangutans use water as a tool	Orangutan	Aesop’s fable	“Köhler (1957) described many of the examples of insight as a perceptual apprehension and recombination of the different parts of a problem […] Here, orangutans produced a solution without seeing the tool (water was inside the drinker), suggesting that they had to think at a more abstract level.”
2009a	Bird & Emery	Insightful problem solving and creative tool modification by captive nontool-using rooks	Rook	Van Bayern paradigm; tool-making; meta-tool-use	“Insight is the ‘sudden production of new adaptive responses not arrived at by trial behavior…or the solution of a problem by the sudden adaptive reorganization of experience’ [(Thorpe 1956)], a concept developed [by Köhler] to explain sophisticated behavior that could not be the result trial-and-error learning.”
2009b	Bird & Emery	Rooks Use Stones to Raise the Water Level to Reach a Floating Worm	Rook	Aesop’s fable	“Seemingly insightful behaviors (see [(Thorpe 1956)] for a definition) …”
2010*	Taylor, Medina, Holzhaider, Hearne, Hunt, & Gray	An investigation into the cognition behind spontaneous string pulling in New Caledonian crows	New Caledonian crow	String-pulling	“Insight has been described as ‘mental scenario building’ where ‘…alternative choices or motor patterns are expressed or suppressed depending on their probable outcome, either before or after such outcome has been experienced’ (Heinrich 2000).” “The ‘insight’ hypothesis claims that this complex behaviour is based on cognitive abilities such as mental scenario building and imagination. An operant conditioning account, in contrast, would claim that this spontaneity is due to each action in string pulling being reinforced by the meat moving closer and remaining closer to the bird on the perch.”
2011	Foerder, Galloway, Barthel, Moore, & Reiss	Insightful problem solving in an Asian elephant	Asian elephant	Use of object to access reward	“The ‘aha’ moment or the sudden arrival of the solution to a problem is a common human experience. Spontaneous problem solving without evident trial and error behavior in humans and other animals has been referred to as insight.”
2011	Hanus, Mandes, Tennie, & Call	Comparing the performances of apes (Gorilla, Pan troglodytes, Pongo pygmaeus) and human children (Homo sapiens) in the floating peanut task	Various great apes, humans	Aesop’s fable	“Results are also consistent with the notion of insightful behavior (Thorpe 1956 and Lethmate 1982).”
2013	Medina Rodriguez	Study of the cognition and its neural substrate in New Caledonian crows	New Caledonian crow	String-pulling	Insight allows animals to build mental scenarios about how to solve novel problems before choosing an appropriate course of action (sensu Heinrich 1995).”
2016	Neves Filho, Bentes de Carvalho Neto, Premi Torres, dos Santos Malheiros, & Christoffersen Knaus	Effects of different training histories upon manufacturing a tool to solve a problem: insight in capuchin monkeys (Sapajus spp.)	Capuchin	Tool-making or meta-tool-use	“’Insight’ can be explained as the spontaneous interconnection of previously acquired behavioral repertoires”; “An insightful solution of a problem is defined as a sudden and new performance, contrasted to a steady, incremental performance like a trial-and-error approach (Hartmann 1933; Thorpe 1956; Chronicle et al. 2001; Ash et al.. 2012).”
2017	Renner, Abramo, Hambright, & Phillips	Insightful problem solving and emulation in brown capuchin monkeys	Capuchin	Aesop’s fable	“the ‘sudden production of a new adaptive response not arrived at by trial behaviour’ (Thorpe 1963), …”
2020	Barrett & Benson-Amram	Can Asian elephants use water as a tool in the floating object task?	Asian elephant	Aesop’s fable	“The floating object task was originally developed to measure insightful problem solving—the sudden production of a new response without trial and error (Thorpe 1963) (i.e., an “ah-ha” moment)”
2022	Sebastián-Enesco. Amezcua-Valmala, Colmenares, Mendes, & Call	Raising the level: orangutans solve the floating peanut task without visual feedback	Orangutan	Aesop’s fable	“… insight (Köhler 1957; Thorpe 1956) …”

(Note that not all of these studies concluded the animal performances were genuinely insightful.)

As can be seen in the table above, the earliest studies of nonhuman animal insight—which predate much of the work on human insight—were performed by Wolfgang Köhler, a founding figure of the Gestalt psychology movement. Köhler was the first person to use ‘insight’ as a term of art for intelligent reasoning that goes beyond the resources of the kind of trial-and-error learning that was thought by most of his predecessors and contemporaries to subsume animal thought. The most prominent of these predecessors was Edward Thorndike (1911), to whom Köhler responds directly and at length. As a Gestalt psychologist, Köhler was also particularly interested in perceptual shifts in an insightful problem-solver’s apprehension of the scene. Köhler describes his famous series of experiments on insight in his 1925 monograph *The Mentality of Apes*: in the most well-known of these, he hung bananas out of reach of chimpanzees, and then provided them with various objects, including sticks and boxes. The chimpanzees were behaving insightfully, he claims, when, after a period of inaction, they suddenly stacked the boxes atop one another to climb them to access the hanging bananas (Köhler 1957). Köhler’s account of insight controlled the narrative for several subsequent decades; his influence can be seen in Table 1.

Today, the dominant characterization of insight in the comparative cognition literature is that of William Homan Thorpe, who was himself greatly influenced by Köhler’s work (see Thorpe 1943). Thorpe (1956) defines insight (or, more accurately, defines what he calls *insight-learning*) as “the sudden production of a new adaptive response not arrived at by trial behavior” or, alternatively, “the solution of a problem by the sudden adaptive reorganization of experience.” As Sara Shettleworth puts it, this definition “is often cited in contemporary publications as *the* definition of insight for animals” (Shettleworth 2012).

Thorpe’s definition is usually understood as specifying a class of behaviors. In certain ways, this is an overly reductive interpretation. Like Köhler, Thorpe was deeply interested in the internal, perceptual state changes (or shifts in *Gestalt*) accompanying moments of insight. His definition of insight makes special note of “the reorganization of experience”, and elsewhere he stresses that insight is fundamentally a matter of “perceptual synthesis” (Thorpe 1974). Nevertheless, this part of Thorpe’s characterization of insight has fallen by the wayside, and, today, he is best known for offering what is thought to be a behavioral operationalization of the concept of insight. Nathan J. Emery, for example, notes that Thorpe’s definition is “based on behavioral criteria”, which “can be applied to any action that is proposed as insightful” (Emery 2013). Emery elaborates:

The important terms to consider here are *sudden*, *new*, *adaptive* and *reorganization of experience*. For an action to be considered the result of insight, it must be spontaneous (i.e., not the result of explicit training or trial and error), novel (i.e., not performed before), functional (i.e., solve the problem and be goal-directed) and built from previous, untrained, similar behavior (i.e., not produced from copying earlier learned responses, but adapting previous behavior into new actions). (Emery 2013).

In this passage, the reader can see how Thorpe’s “reorganization of experience” criterion is construed merely as requiring a behavior to be “built from previous, untrained, similar behavior”, an interpretation that obscures Thorpe’s interest in complex perceptual reorganization but which has the benefit of making insight a straightforwardly observable phenomenon.

The second thing to note is that by stipulating that insightful behavior must not be the result of training, trial-and-error, or copying, Thorpe and his interpreters are, first and foremost, attempting to rule out cases where the behaviors are the products of ‘mere association’, *i.e.*, stimulus-bound and autonomic processes performed as the result of prior conditioning. Indeed, the elimination of purely associative explanations for putatively insightful behaviors is quintessential to work on insight in nonhuman animals. In contrast to the relatively nuanced understanding of insight developed in work on human psychology, those working on insight in nonhuman animals *predominantly* characterize insightful problem-solving as simply the counterpart to less ‘cognitive’ or ‘rational’ forms of problem-solving that proceed by way of automatic mechanisms. An example of this can be found in Bird and Emery (2009a, b), which glosses the Thorpean definition of insight as “a concept developed to explain sophisticated behavior that could not be the result trial-and-error learning”; numerous other examples appear in the table above, including Bernd Heinrich’s 1995 study of insight in ravens, which defines insight as that which “can be shown indirectly to play a role in a behavior where learning and/or responses present from birth can be eliminated.”

Given the comparative use of insight as a conceptual contrast to associative mechanisms, the bulk of the debate in comparative psychology surrounding alleged demonstrations of insight in nonhuman animals concerns whether we can truly rule out fully associative accounts of the behaviors in question. Consider, for example, Köhler’s box-stacking experiment, mentioned earlier. A classic study by Epstein et al. (1984) showed that pigeons could be induced to solve a similar box-stacking problem using only the tools of associative learning, and furthermore that their behaviors appeared very like those of Köhler’s chimpanzees. With his experiment, Epstein raised the worry that has subsequently dogged studies of insight in nonhuman animals, namely that observers may be unable to distinguish genuine cases of insightful problem-solving from products of mere associative learning.^2^ More recently, Taylor et al. (2012) used New Caledonian crows to raise similar doubts about much contemporary work on avian insight (including his own). As we increasingly appreciate the extent to which associative mechanisms can drive behavior, some have worried that it might be the case that no putative cases of insight in nonhuman animals is immune from debunking.

I think we need not be quite so pessimistic. There are cases of insight in nonhuman animals that do appear to be sufficiently non-associative so as to satisfy the Thorpean criteria, some of which are especially robust in that a great deal is known about the previous learning histories of the nonhuman animals in question. One class of experiments that seems to withstand this worry particularly well is the Aesop’s fable paradigm, wherein subjects must obtain a floating reward from a deep, narrow container by adding water to the volume or displacing the existing water with stones. This is in contrast to typical direct approaches, such as reaching for the reward with fingers, beaks, or claws, which are insufficient to the task.^3^ Species that have successfully solved this problem in controlled experimental settings include chimpanzees (Hanus et al. 2011), orangutans (Mendes et al. 2007), and rooks (Bird and Emery 2009b). Many of these experiments provide strong evidence for insightful problem-solving in the Thorpean sense, given the novelty of problem, the novelty of the application of the ‘insightful’ solution behavior (such as spitting water into the volume) to the present class of objects and stimuli, and the suddenness of the emergence of the ‘insightful’ solution after failed direct attempts (though often still during the first session of exposure to the problem) (Shettleworth 2012).^4^ Furthermore, although I will not discuss them here, some other experimental paradigms have also provided similarly robust evidence of insight in nonhuman animals, particularly those involving the construction and use of novel tools (Bird and Emery 2009a; Emery 2013; Shettleworth 2009).

So, it seems that the failure of some putative cases of animal insight to satisfy Thorpe’s criteria is not in itself necessarily an ill omen for the possibility of a successful comparative study of insight, as there remain a class of cases that *do* plausibly satisfy the criteria. At the very least, I think we can grant, if only for the sake of argument, that comparative psychologists do have a working definition of insight that does succeed in picking out a number of nonhuman animal performances as insightful. To suggest that we cannot mount a successful comparative study of insight in humans and nonhuman animals because there are no nonhuman animals that genuinely demonstrate Thorpean insight would, then, be premature. However, as I will show, it would be equally premature to treat, as some have done, the existence of cases that do meet this bar as a proof of concept for the present legitimacy that enterprise.

## Arbitrary disjunction

In Sect. "Can animal insight teach us about ourselves?", I set out three necessary conditions for a successfully comparative study of insight:

1. We must be able to identify cases of insight in humans.
2. We must be able to identify cases of insight in nonhuman animals.
3. These cases of insight must plausibly be exercises of the same capacity, based on criteria according to which insight is not arbitrarily disjunctive.

Section "Insight in Humans and nonhuman animals" demonstrated the requirements set out in (1) and (2) are nominally satisfied, and explained how the concept of insight has been defined and operationalized in the study of both humans and nonhuman animals. However, in this section I will argue that the *manner* in which conditions (1) and (2) are satisfied raises serious worries about the likelihood of satisfying condition (3).

First, note the stark differences between the methodologies deployed in studying insight in humans versus nonhuman animals. To illustrate, I will directly compare a human who demonstrates insight by solving the nine-dots problem with a nonhuman animal who demonstrates insight by solving the Aesop’s fable paradigm’s water displacement problem. In both cases, the problem-solvers behaviorally demonstrate an awareness of the solution to the problem, either by drawing the correct series of strokes in the case of the nine-dots problem or by cleverly using water to obtain a reward in the case of the Aesop’s fable paradigm. Although these insightful performances have a great deal in common, there is also much that is distinct, not least of which is that in the human case there is verbal testimony available as to the experiential character of the problem-solving exercise and that in the nonhuman animal case there is not. Furthermore, the nature of the tasks themselves are often quite different. An insightful solution of the nine-dots problem typically involves the reinterpretation of a stated rule’s scope of application. By contrast, the Aesop’s fable paradigm is primarily a test of causal or means-end reasoning, and the insight that its solution requires involves the exercise of those reasoning abilities in combination with an awareness of certain physical or environmental facts (*e.g.*, the properties of fluid in a volume).

This itself is not necessarily a problem. It is not unusual that the test for the presence of some capacity in nonhuman animals should differ radically from that used with humans. As long as the underlying capacity in question is well-understood, and the performance measures for insight in humans and nonhuman animals plausibly pick out the self-same capacity, then the successful comparative study of insight might be achieved. This is what is meant by the requirement that what makes something count as an instance of insight must not be arbitrarily disjunctive. If we are to credit the human who solves the nine-dots problem and the nonhuman animal who solves the Aesop’s fable task with the same kind of achievement, it is crucial that insight is *best* understood as a singular capacity (or possibly a unified suite of capacities) that is exercised by *both* humans and nonhuman animals, and not as the exercise of one of two fundamentally different capacities, one that is exercised by humans and one that is exercised by the nonhuman animals.

To illustrate, let us consider a well-understood perceptual capacity that easily satisfies this prohibition against arbitrary disjunction, namely the capacity for vision. The capacity for visual perception is enjoyed by humans as well as many nonhuman animals, for example serpents. Although there are many notable physiological differences between human and, *e.g.*, reptilian visual systems, and empirical measures for assessing the visual capacities of both vary, we nevertheless easily appreciate that ‘vision’ as applied to humans and ‘vision’ as applied to serpents pick out same functional capacity. This makes it possible for us to engage in all manner of meaningful comparative work about vision in general.

Contrast the case of vision with what I shall call “visi-sodic memory”, a fictional ‘capacity’ which I have invented for the sake of this argument. Let something count as an exercise of visi-sodic memory if and only if it is *either* an exercise of vision in humans *or* an exercise of episodic memory in nonhuman animals such as serpents. This is an example of a capacity that would be deemed *arbitrarily disjunctive*. It should be self-evident that the study of visi-sodic memory on the basis of the differences between its manifestations in humans and nonhuman animals would not be worth pursuing for the sake of any legitimate comparative end (at the very least, not one that is not far better characterized as in pursuit of understanding some other far more general capacity to which both vision in humans and episodic memory in serpents are relevant). If we hope to succeed in the comparative study of insight across both humans and nonhuman animals, then, we must be sure that insight is more akin to vision than visi-sodic memory_._

One reliable litmus test for capacities that are arbitrarily disjunctive is that they are difficult to articulate in species-neutral terms. Vision, which is not arbitrarily disjunctive, is easily describable in species-neutral terms as the capacity to process a certain class of perceptual stimuli. The arbitrarily disjunctive visi-sodic memory, in contrast, can most easily be described as the capacity to process visual stimuli if one is a human and to engage in a certain kind of conscious recollection if one is a nonhuman animal. A more species-neutral characterization is hard to devise. Humans who exercise visi-sodic memory may be incapable of episodic memory, and nonhuman animals who exercise it might very well be blind.

I contend that insight, while not quite as egregiously as visi-sodic memory, is also arbitrarily disjunctive. It is not possible to describe insight as a species-neutral capacity such that the tests that have been used to identify it in humans and those that have been used to identify it in nonhuman animals are plausibly tracking the same thing. Not only are those who study insight in humans and nonhuman animals potentially identifying exercises of vastly different capacities in their respective inquiries, the theoretical reasons that each of them are *interested* in the study of insight are very different, which presents obstacles to either party revising their conception of insight to better harmonize with that of the other.

For this reason, the Thorpean criteria cannot serve as the foundation of a cross-species study of insight that also captures what we talk about when we talk about insight in humans. Likewise, more anthropocentric understandings of insight cannot meaningfully be applied to nonhuman animals. This will become increasingly apparent in the remainder of this paper, in which I shall raise three increasingly serious problems for the comparative study of insight in humans and nonhuman animals. First, the Thorpean stipulation that insightful behavior must be non-associative is out of step with the current literature on insight in humans. Second, Thorpe’s criteria for insight are too permissive: there is a broad set of clearly non-insightful behaviors in humans they would categorize as insightful. Third, even if we could test for insight’s distinctive phenomenology in nonhuman animals, there is little reason to think it would be present in most cases. Rather, most ‘insightful’ nonhuman animal performances are better understand in terms of other high-level cognitive capacities, such as instrumental reasoning.

## Problem one: the non-associative constraint

As I discussed previously, on the comparative understanding of insight, insightful problem-solving has always been understood as necessarily involving something over and above associative processing. Indeed, no quality is seen to be more fundamental in that literature, where arguing against the interpretation of a behavior as insightful typically *just is* to explain it in terms of ‘mere’ associative processes. If those who use the Thorpean criteria to study insight in nonhuman animals are truly talking about the same capacity as those who study insight in humans, it had better make sense, then, that insight in humans, too, cannot be the result of purely associative processing. Yet those who study insight in humans no longer consider this an uncontroversial assumption. The associative theory of creativity, for example, holds that insightful solutions are formed via a series of associative processes in semantic memory (Bowden et al. 2005; Kounios and Beeman 2014; Mednick 1962). Our lack of conscious access to the processes underlying insight (Metcalfe and Wiebe 1987) is also suggestive that that associative processes may be doing much of the work. At the very least, more must be said about why comparative psychologists working with nonhuman animals, contrary to those working with humans, so widely believe that a process whose intermediate stages are phenomenologically inaccessible to humans must be non-associative in animals, particularly given that such inaccessibility is in many ways more characteristic of associative than non-associative processing.

One can put it in terms of the dual-process model’s distinction between System 1 and System 2, in which the mental processes of System 1 are fast, associative, and non-conscious while those of System 2 processing are slow, consciously accessible, and goal-directed (Kahneman 2011). While ordinary analytic reasoning is at home in System 2, the features of insight seem more characteristic of System 1. Indeed, some psychologists have explicitly argued insight is a System 1 phenomenon; others that it is, at the very least, an interesting intermediary process between the two systems (Lin and Lien 2013; Sowden et al. 2015).

In light of this, it might seem somewhat surprising that comparative psychologists persistently discuss insight as if it were a particularly robust System 2 process, and moreover a marker of deliberative intelligence in nonhuman animals. The reasons for this are partly historical. The first thing to note is that those studying insight in humans and in nonhuman animals did not always differ in opinion about whether insightful problem-solving could be wholly attributable to associative processes. During the first half of the twentieth century, it was the standard assumption in the study of insight in *both* humans and nonhuman animals that it was a non-associative phenomenon (Patton 1932). Furthermore, some of the earliest work on insight in humans was highly collaborative with work on nonhuman animals, partly due to the influence of Wolfgang Köhler, mentioned earlier. The concepts of insight deployed in work on humans and work on nonhuman animals only began to diverge in the latter half of the twentieth century, when an increasing number of cognitive psychologists studying insightful problem-solving in humans entertained the idea that conventional (and sometimes associative) learning mechanisms might play a central role in explanations of insight, and even perhaps creativity more generally (Ginsburg and Hood 1970; Mednick 1962; Sternberg and Davidson 1995). At the same time, during many of these developments, the study of insight in nonhuman animals had fallen relatively dormant, and thus few comparative psychologists were in a position to respond to these sea changes in cognitive psychology. When the study of insight in nonhuman animals experienced its later resurgence, particularly in the mid-nineties, the comparative psychologists picked up more or less where they had left off, without having revised their working understanding of insight to reflect the theoretical developments that had taken place in the interim. “The comparative literature,” Josep Call has lamented, “could have benefited from insights from cognitive psychology in this area, but such transfer of ideas has not taken place, at least not as much as it occurred many years ago” (Call 2013). Thus, by taking for granted that insight is non-associative, comparative psychologists now beg the question on something that is very much now a matter of debate.

To be clear, I do not think this worry alone constitutes an insurmountable obstacle. There remain researchers working on insight in humans who *do* still think insightful problem-solving is (at least partly) a non-associative, System 2 process, and who have cogent reasons for thinking so (Chu et al. 2007; Gilhooly and Murphy 2005; Gilhooly and Webb 2018; MacGregor et al. 2001; Mayer 1995). Thus, although the Thorpean concept of insight is in tension with the way some psychologists understand insight in humans, perhaps a successful collaborative research program could still emerge between the former and those psychologists who agree that insight is non-associative, barring other obstacles.

## Problem two: Thorpe’s criteria are too permissive

A second, deeper worry for the reconcilability of the human and nonhuman concepts of insight is that there are some human behaviors that are obviously not the products of insightful problem-solving, but which nonetheless satisfy Thorpe’s criteria for insight, and would, therefore, in a nonhuman animal, likely be classified as insightful in a nonhuman animal. Consider the following case, based loosely on Köhler’s box-stacking experiment:

Although I have historically been exceptionally tall, a recent back injury has left me with a pronounced stoop, and I now walk with a cane. One day, while cooking breakfast, I go to retrieve a box of pancake mix from the pantry. The box is on the highest shelf. Though I could reach items on that shelf before my injury, when I attempt to do so now, I discover that the box is far beyond my reach, even when I stand on my toes. “If I want to reach the pancake mix,” I think, “I will, at the very least, need something to stand on.” After some deliberation about what tools might best serve my needs, I fetch a chair from the dining room. Balancing precariously atop the chair, I can just barely reach the pancake mix with the end of my cane. I knock down the box, retrieve it, and commence with breakfast.

This case satisfies all of Thorpe’s criteria for insightful problem-solving. I fetch the chair only after my habitual methods of reaching the pancake mix have failed. We can stipulate that my use of the chair is genuinely novel in the context of my behavioral history, as I have never before needed to use a chair or a cane to extend my reach in this manner. If you like, we might also (somewhat more fancifully) stipulate that this is not even something that I have previously observed other people doing. My solution was the product of at least some cognitive processing, as I reached it through occurrent deliberation. And, once I committed to that solution, I executed it in its entirety, smoothly and rapidly. By the lights of an observer applying Thorpe’s criteria, then, I appear to have demonstrated insight. But to cognitive psychologists studying insight in humans, and indeed to anyone who is at all familiar with what it’s like to experience episodes of insightful problem-solving, my solution to the problem of the pancake mix is obviously not an insightful one.

Firstly, in the example above there is no *experience* of breaking through a psychological impasse. I never took myself to be unable to solve the problem before me, and although my first attempt to reach the pancake mix was a failure, at no point did it seem to me as if there were no possible means of obtaining it. Nor did I, open forming my plan, experience the ‘aha!’ phenomenology that traditionally accompanies flashes of insight.

Secondly, my apprehension of the problem’s solution does not involve cognitive restructuring of the kind common to insightful solutions. My initial representation of the problem space did not explicitly include the chair and my cane among its elements, but neither were they excluded by some assumption. The moment that reaching the pancake mix became a problem that I needed to solve, it became apparent that I required the assistance of objects of that nature in order to solve it.

It is clear that merely applying Thorpe’s criteria for insight to human performances will lead to a proliferation of ‘false positives’. A more robustly unifying notion of insight is needed if we are to informatively study it in both humans and nonhuman animals. Might we reconcile the Thorpean definition of insight in nonhuman animals with the cognitive psychologists’ definition of insight in humans to arriving at a hybrid definition that can be probatively applied to both? The differences in the criteria used to identify insight in each, and even the fact that there are cases in which these criteria render different verdicts about whether a performance is insightful, are not *necessarily* defeating. So long as there were *enough* overlap between the behaviors deemed insightful in humans and those deemed insightful in nonhuman animals, it might still be plausible that both groups of researchers are, in at least a plurality of cases, latching on to the same phenomenon. In other words, if there exists some identifiable subset of human *and* nonhuman animal behaviors that both parties are willing to agree to call insightful, then fruitful collaborations about the nature of insight across species remain possible.

Unfortunately, I suspect that there exists no stable subset of human and nonhuman animal behaviors that both parties would recognize as insightful. Although comparative psychologists do already eagerly equate insightful problem-solving in humans to the behaviors they are studying in nonhuman animals, cognitive psychologists are unlikely to share these universalist proclivities.

Even if the comparative psychologists were willing to accept all of the commonly cited human performances of insight as genuinely insightful, there is likely no extant case of animal insight (l*et al.*one subset of such cases) that the cognitive psychologists studying insight in humans would likewise agree to accept with the same level of certainty. This is because, as I have stressed, according to their methodological framework, the distinctive phenomenological profile of insight is not just a central diagnostic criterion but an indispensable one. The pancake mix case above does not seem to be a valid case largely because insight’s distinctive phenomenology is absent. Putative cases of nonhuman animal insight face the issue that a creature’s satisfaction of Thorpe’s criteria does not in itself entail anything about that creature’s phenomenal states. Quite simply, we lack the means of reliably identifying the occurrence of ‘aha!’ phenomenology in nonhuman animals.

This is not to say we have no general insight into the phenomenological states of nonhuman animals; a look at recent work studying consciousness, pain, and affective states in nonhuman animals shows otherwise (Birch et al. 2020; Paul et al. 2020; Sneddon et al. 2014). Yet this is still a growth area for comparative psychology and the literature concerning affective states in particular is not yet at a stage where it can be of service in the identification of insight, even with the best observational, experimental, and neuroscientific methods currently available. This is because distinguishing insight’s characteristic phenomenology from other phenomenological states is no easy task. For example, it is difficult to distinguish the behaviors of a creature experiencing the excitement of solving a problem (and gaining access to a reward, say) and a creature that is experiencing the realization of a genuinely insightful solution, especially given that the latter is often also accompanied by the former.

What if we could tap into nonhuman affective states with the specificity necessary to identify insight’s distinctive phenomenology? Might we then make progress? Perhaps. However, in the next section, I argue that even if we could test for insight phenomenology across the species, we would discover it not to be present in many of the most commonly proposed instances of insightful problem-solving in nonhuman animals.

## Problem three: alternative explanations

Even if we *could* test for the distinctive phenomenology of insight in nonhuman animals, there is little reason to think it would be present in most of the putative cases of animal insight picked out using the Thorpean criteria. Whereas, in the previous section, I argued that putative cases of nonhuman insight, whether genuine or no, struggle to discharge the burden of proof in the absence of information about nonhuman phenomenological states, in this section, I argue that the behaviors in question are unlikely to be instances of insight at all, and that better accounts of ‘insightful’ nonhuman animal behaviors will place the explanatory burden on the exercise of cognitive capacities that are not distinctively related to insight.

Many alleged instances of insightful problem-solving in nonhuman animals may simply reflect other cognitive distinctions, such as a sensitivity to (or, more ambitiously, an understanding of) causal relationships. Perhaps when initial, automatic efforts to solve a problem fail, nonhuman animals, if they can do so, are more likely to shift their attention to the causal relationships within the problem space, as humans in the same situation often do (Tomasello and Call 1997). Though these shifts in attention may make the solution to a problem newly accessible, they are not particularly *insightful*. Rather, they are simply the result of the methodical (and perhaps even the deliberate) application of a higher-level mental capacity after lower-level capacities have failed to make headway. This is far more similar to the stepwise problem-solving deployed in the pancake mix case in the previous section than to anything cognitive psychologists would recognize as insight. The human in the pancake mix case does not say “aha”—neither, were they so able, would many putatively insightful nonhuman problem-solvers.

Successes on the Aesop’s fable paradigm, at string-pulling tasks, and in many tool-use experiments are certainly best interpreted in this way. Indeed, some researchers who have run these experiments have left insight out of their discussions altogether,choosing to interpret their subjects’ behaviors only as demonstrations of causal reasoning (e.g., Davidson et al. 2017; Stanton et al. 2017). Others have spoken out explicitly *against* the insightful interpretation of such behaviors, such as Sarah Jelbert and colleagues, who argue, concerning the Aesop’s fable paradigm, that, “[w]hile success on [the Aesop’s fable] task is unlikely to represent an insightful solution, [the paradigm] does provide a useful avenue to explore causal reasoning and physical cognition in animals” (Jelbert et al. 2015). Von Bayern et al. (2009) write that, “[i]n our opinion, ‘insight’ can never be a satisfactory explanation for an animal’s innovative performance because this label avoids identifying the exact processes by which a solution is obtained.” Kenward et al. (2006) call the concept of insight “problematic” and use it only “for its heuristic value”.

Even researchers who *do* think that successes at the Aesop’s fable task and similar tests demonstrate insight often consider the insights that are reached to be *insights about causal relationships*—and so the capacity for causal understanding is already baked into even their explanations, and arguably doing most of the heavy lifting. According to Kenward et al*.* (2006), for example, the sense in which tool-using New Caledonian crows are insightful amounts to their being “equipped with cognitive mechanisms that allow [them] to learn physical laws by observing object interactions, and then plan goal-directed tool-oriented behaviour that exploits these laws”. Similarly, Sebastián-Enesco et al. (2022) write:

Insight … [relies] necessarily on some understanding of the causal relations involved in the problem that is activated before acting, whereas sensorimotor mechanisms mainly depend on the feedback generated by physically acting on the problem.

Werdenich and Huber (2006), as can be seen in Table 1, while not opposed to calling their keas insightful, likewise emphasize the role of causal understanding, and connect their findings far more extensively to the literature on causal cognition in humans than that devoted to insight.

Other higher-level cognitive capacities, too, are often recruited to explain ‘insightful’ nonhuman animal behaviors, such as imaginative first-person simulation or the visualization of how objects might fit together in the scene (Finke and Slayton 1988; Gruber et al. 2019; Redish 2016). Like causal cognition, many of these capacities have well-established comparative research programs.

Perhaps the richest possible reinterpretation of ‘insight’ in nonhuman animals understands it in terms of means-end (or instrumental) reasoning, which involves not just causal cognition but also goal-directedness (Camp and Shupe 2017). A creature has the capacity for means-end reasoning if they are able to identify and execute intermediary courses of action in service of some larger goal. Critically, they must also grasp that those intermediary courses of action are appropriate *because* they centrally contribute to actualizing that goal.

This capacity for means-end reasoning is an important cognitive milestone. In humans, it develops in infancy, around 6 or 7 months of age, and competence grows until the infant can engage in spontaneous and fully intentional means-end actions between 9 and 12 months (Piaget 1952; Sommerville and Woodward 2005; Willatts 1999). Means-end reasoning is also an important phylogenetic milestone, and there is a rich literature in comparative psychology on its evolutionary emergence and cognitive role (see Krasheninnikova 2022 for a general overview).

In previous sections, I made the case that the different understandings of insight used in the literatures on humans and nonhuman animals are in tension with one another. I argued that the widespread Thorpean criteria for recognizing insight in nonhuman animals is particularly ill-suited to tracking the features of insight most of interest to psychologists working with humans, and is perhaps even unable to reliably distinguish between insightful and non-insightful problem-solving. At a minimum, I take my criticisms to support the conclusion that the Thorpean understanding of insight can do little productive comparative work and falls short of what is needed to fruitfully connect research on insight in nonhuman animals to the study of insight in humans. Yet the fact that we can readily reinterpret ‘insightful’ animal behaviors as exercises of other higher-level cognitive capacities and interpret them within the broader framework of means-end reasoning should be a consolation to the comparative psychologist. Far from being wasted work, the bulk of experiments on insight in nonhuman animals can be retrofitted into the existing and closely related comparative research program devoted to the development and exercise of means-end reasoning in humans and nonhuman animals.

This move away from insight and towards other capacities such as means-end reasoning is less revisionary than one might expect. Many researchers who deploy the standard experimental paradigms that appear in work on nonhuman animal insight actually already connect their work to “insight” only minimally or not at all, thus sidestepping the pitfalls I have outlined in this paper (see, for example, Gagne et al. 2012; Gruber et al. 2019; Heinrich and Bugnyar 2005; and Taylor et al. 2014). Additionally, many are already sensitive to the contributions of means-end reasoning and its ancillary capacities to ‘insightful’ animal performances (Cook and Fowler 2014; Foerder et al. 2011; Huber and Gajdon 2006; Kenward et al*.* 2006; Kirsch et al. 2008; of Menie et al. 2021; Sebastián-Enesco et al. 2022).

In the experimental literature on nonhuman animals, the role that insight is most frequently called upon to serve is that of a mere foil to simpler, associative explanations of animal behavior; but, as I have shown, insight does not play this role in the study of human psychology, where it is of interest largely because of its role in creative thought and its distinctive phenomenological profile. Moreover, psychologists disagree about whether the processes culminating in human insight are best understood as associative or non-associative processes.

Sara Shettleworth (2010) has argued that researchers who ask whether nonhuman animals are insightful are guilty of making a category mistake, investigating what amounts to a folk psychological concept as if it were a discrete cognitive capacity. Instead, she argues, these researchers should identify the cognitive mechanisms *underlying* episodes of insightful problem-solving, only some of which are likely to be attributable to nonhuman animals, and proceed from there. Shettleworth illustrates the strategy she has in mind:

An outstandingly successful example of this approach is the study of language evolution, in which the old question, ‘Can animals learn language?’ has been replaced by appreciation that although human language is just that, human, other species share important components of it. For instance, highly social primates may have evolved hierarchical conceptual abilities contributing to language, and neural and developmental control of songbirds’ vocal learning is instructively analogous to that of humans. (Shettleworth 2010).

Like Shettleworth, I believe we ought to refocus our investigations into nonhuman animal insight on its various component capacities—and I go somewhat further in my claim that the capacity that is *most* relevant to the interests of those engaged in the literature is means-end reasoning. One reason for this is that means-end reasoning is particularly well-suited to serve, as nonhuman insight has done, as a contrast to associative, trial-and-error learning and problem-solving. One of the distinctive features of means-end reasoning is that it is *not* stimulus-bound, or, in other words, merely associative—otherwise, it would not be a form of reasoning at all. As Camp and Shupe have articulated it,

[instrumental, or means-end, reasoning] stands as a landmark on a trajectory from simple stimulus-response association to purely theoretical deliberation. A creature who can reason instrumentally doesn’t just respond directly to its immediate environment, as a mouse fleeing the scent of a cat does. Nor does it act directly to satisfy a need, like a hungry bird flying off to a cache of nuts. Instrumental reason severs the direct connection between representation and action by interposing a representation of a possible state. (Camp and Shupe 2017).

To illustrate the point with an example of a behavior that is not limited by its proximity to stimulus, consider Osvath’s and Karvonen’s 2012 account of a chimpanzee concealing projectiles under piles of hay in order to later throw them at zoo visitors. This behavior suggests to Osvath and Karvonen that “chimpanzees can represent the future behaviours of others while those others are not present, as well as take actions in the current situation towards such potential future behaviours.” Furthermore, and importantly, “the behaviour of the chimpanzee produced a future event, rather than merely prepared for an event that had been reliably re-occurring in the past”, further distancing it from the cycle of behavioral adaptation following direct observation (Osvath and Karvonen 2012).

Thus, in the comparative cognition literature, not only is talk of means-end reasoning in nonhuman animals able serve much the same purpose talk of insight currently serves, its importance as a stepping stone towards high-level, rational inference also allows it to be used as a foil for associative processes in much the same way that insight has been used previously.

Were our understandings of insight in humans and nonhuman animals more compatible, discoveries about insight in nonhuman animals would have had the potential to constrain our theories about the cognitive underpinnings of insight in humans. Given that this does not seem to currently be possible in the case of insight, but might be possible in the case of means-end reasoning, what can extant work on ‘insight’ in nonhuman animals, so reinterpreted, teach us about means-end reasoning in humans?

Potentially, a great deal. Evidence of ‘insightful’ means-end reasoning in avian species, for example, allows us to draw exciting conclusions about the evolutionary pressures behind its emergence in humans. Given that birds phylogenetically diverged from mammals 300 million years ago (Laurin and Reisz 1995), birds (and, potentially, more distantly related taxa) can tell us far more about the origins and prerequisites of such capacities than our fellow primates can. Specifically, ‘insightful’ string-pulling and Aesop’s fable successes in avian species suggests that the neocortex may not be necessary for means-end reasoning. Kirsch et al. (2008) provides a proof of concept of this approach, arguing that several “insight-related cognitive functions”, including causal reasoning, are, for this reason, “not necessarily based on a laminated cortical structure” but can rather “be generated by differently organized forebrains.” By decomposing insight into its ancillary capacities, Kirsch and her co-authors connect avian insight experiments not only to the causal cognition literature but also to those devoted to object permanence, theory of mind, and mental time travel (Kirsch et al. 2008).

## Conclusion

In conclusion, the way psychologists understand and operationalize insight varies depending on whether they are studying the phenomenon in humans or nonhuman animals. Given the tension between its respective roles in these literatures, the prospects for developing a successfully comparative characterization of insight, according to which its criteria of application are not arbitrarily disjunctive, are somewhat dim. Furthermore, the theoretical reasons that insight is of interest to those who study it in humans and those who study it in nonhuman animals are radically different, leading to further challenges for intellectual collaboration.

Why not set ‘insight’ in nonhuman animals aside, then, and more parsimoniously let causal cognition, means-end reasoning, and other ancillary capacities do the bulk of the explanatory work, as many researchers already seem inclined to do?

## Data Availability

This declaration is not applicable.

## Footnotes

1 Though I use the name used by Neilands et al. (2016) for this paradigm, note that Von Bayern (2009) credits Bird & Emery (2009a) with developing this task.

2 Although I will not go into the details of Epstein’s experiment here, Emery (2013) provides some commentary: “What about Epstein’s pigeons? Their behavior was not spontaneous (it was based on training), not functional (no reward), not novel (pecking and pushing – a form of peck – are both in a pigeon’s repertoire) and was not adapted from previous similar behavior (rather than previous same behavior), so could not be considered as insight in any sense.” A follow up experiment with pigeons by Cook and Fowler (2014) has also used appropriate controls to conclusively show that the driving mechanisms here are purely associative.

3 The paradigm is named for Aesop’s fable of the Crow and the Pitcher, where a thirsty crow drops stones into a pitcher of water to cause the water level to rise high enough to allow him to drink.

4 Note, however, that there are, as always, researchers who remain critical; see Jelbert et al. (2015), which I will return to later in this paper, for a skeptical explanation of these performances in rooks as merely prompted by rewarding perceptual feedback.
